# Estrogen-induced SDF-1α production promotes the progression of ER-negative breast cancer via the accumulation of MDSCs in the tumor microenvironment

**DOI:** 10.1038/srep39541

**Published:** 2016-12-20

**Authors:** Liquan Ouyang, Weilong Chang, Bin Fang, Jieting Qin, Xincai Qu, Fanjun Cheng

**Affiliations:** 1 Institute of Hematology, Union Hospital, Tongji Medical College, Huazhong University of Science and Technology, Wuhan 430022, China; 2 Department of General Surgery, The First Affiliated Hospital of Zhengzhou University, Zhengzhou 450052, China; 3 Department of Clinical Medicine, Yichang Hospital of Traditional Chinese Medicine, Hubei University of Chinese Medicine, Yichang 443000, Hubei, China; 4 Department of Breast and Thyroid Surgery, Union Hospital, Tongji Medical College, Huazhong University of Science And Technology, Wuhan 430022, China

## Abstract

Estrogen plays a role in the processes of tumorigenesis, metastasis, and drug resistance in estrogen receptor (ER)-positive breast cancer (BC). Whether estrogen contributes to ER-negative BC is unclear. Here, we aimed to investigate whether estrogen could stimulate the secretion of stromal-derived factor-1 (SDF-1α) by cancer-associated fibroblasts (CAFs) to promote the progression of ER-negative BC. We transplanted ER-negative BC cells into ovariectomized mice, which was followed by continuous injection of estrogen, and found that estrogen promoted the tumorigenesis of BC. Furthermore, High levels of SDF-1α and tumor-infiltrating myeloid-derived suppressor cells (MDSCs) were detected in the estrogen treatment group. Estrogen stimulates secretion of SDF-1α by CAFs extracted from BC patients. Recombinant SDF-1α could recruit MDSCs isolated from bone marrow cells of mice. In addition, the co-culture of CAFs and MDSCs demonstrated that the recruitment of MDSCs was increased when CAFs were exposed to estrogen. Using AMD3100 to block the SDF-1α/CXCR4 axis or gemcitabine to delete MDSCs, we observed that both of these agents could neutralize the effect of estrogen on tumorigenesis. Together, these results suggest that estrogen may promote the progression of ER-negative BC by stimulating CAFs to secrete SDF-1α, which can recruit MDSCs to the tumor microenvironment to exert tumor-promoting effects.

Breast cancer (BC) is the most common malignancy among women. Approximately 75% of breast cancers are estrogen receptor-positive and belong to the luminal molecular subgroup. The most widely accepted theory of how estrogen influences estrogen receptor (ER)-positive BC is that estrogen stimulates tumorigenesis, metastasis, and resistance to endocrine therapy in human breast cancer by acting through the ER. However, ER-negative breast cancer accounts for a certain proportion of the total number of breast cancer cases, and patients with this BC subtype always have a poor prognosis. Whether estrogen contributes to ER-negative BC remains unclear. Studies have suggested that ovariectomy prevents the development of both ER-positive and ER-negative BC^1^,^2^,^3^, which indicates that even the progression of ER-negative BC may depend on ovarian hormones. In a reported experimental model, estrogen increased the metastasis of ER-negative murine 4T1 cells^4^. Recent studies have shown that estrogen may affect ER-negative BC through systemic effects on the host compartment and not by direct action on tumor cells^5^,^6^. Some investigators have found that estrogen can promote the metastasis of D121 Lewis lung carcinoma cells and the tumorigenesis of PC-3 human prostate cancer cells, which lack ERα/β expression. These studies indicate that estrogen may influence ER-negative BC via an indirect mechanism and not via the classical estrogen-dependent pathway.

The tumor microenvironment plays an important and intricate role in the progression of BC. Cancer stromal cells include endothelial cells, immune cells, and fibroblasts, which are the most abundant cell type in the tumor microenvironment. Cancer-associated fibroblasts (CAFs), which are known as active stromal cells, play important roles in influencing tumor progression. The expression of estrogen receptor α in cancer-associated fibroblasts can suppress the invasiveness of prostate cancer, which indicates that estrogen can influence cancer progression via stromal cells in the local microenvironment^7^. Moreover, G protein-coupled estrogen receptors (GPERs) have recently been reported to be biomarkers in estrogen-related cancers. The action of these receptors is different from that of the classical nuclear estrogen receptors^8^. A large amount of evidence has revealed that GPERs are exclusively expressed as a type of ER in mammary CAFs and that signaling via these receptors induces the expression of several downstream genes in response to estrogen, which results in the promotion of proliferation and metastasis^9^,^10^,^11^. Furthermore, CAFs, which exhibit the traits of myofibroblasts, play a significant role in the promotion of growth and angiogenesis of tumor cells through their ability to secrete various extracellular matrix components, such as collagen, proteoglycans, proteases, growth factors, cytokines, and chemokines (e.g., stromal cell-derived factor 1 (SDF-1α))^12^. SDF-1α (known as CXCL12) is a member of the CXC chemokine family and is involved in cell migration and leukocyte chemotaxis, which promote proliferation and metastasis in many cancers^13^. Recently, SDF-1α was identified as an estrogen-regulated gene in ER-positive ovarian and BC cells^14^, but the induction of the expression and secretion of SDF-1α by estrogen-stimulated CAFs has not been reported.

SDF-1α is produced by stromal and tumor cells in the tumor microenvironment. It exerts multiple tumor-promoting functions via either its receptor CXCR4, which is expressed on cancer cells and enhances tumor growth and metastasis, or the recruitment of endothelial progenitors for tumor angiogenesis. SDF-1α can also recruit some immunosuppressive cells, such as regulatory T cells and dendritic cells, to the tumor environment, where they play a crucial role in immune evasion and limiting the effectiveness of the immune response^15^,^16^. MDSCs are a heterogeneous population of immature myeloid cells that cause tumor-associated immune abnormalities and contribute to tumor-mediated immune escape. Local SDF-1α and its receptor CXCR4, which is expressed on tumor-associated MDSCs, participate in the recruitment of MDSCs to the tumor environment^17^. Thus, it is possible that CAFs within breast tumors exert tumor-promoting effects largely through the secretion of SDF-1α, which acts through not only the cognate receptor CXCR4 on cancer cells but also the recruitment of MDSCs for immune escape.

In the current study, we established an experimental murine model to investigate the effect of estrogen on ER-negative BC. We found that estrogen stimulated CAFs to produce SDF-1α, which recruits MDSCs into the tumor microenvironment, where they exert tumor-promoting effects. Inhibition of the recruitment of MDSCs can reverse the tumor-promoting effects of estrogen in ER-negative BC.

## Results

### Estrogen promotes the tumorigenesis of estrogen receptor-negative breast cancer

**Figure 1:**
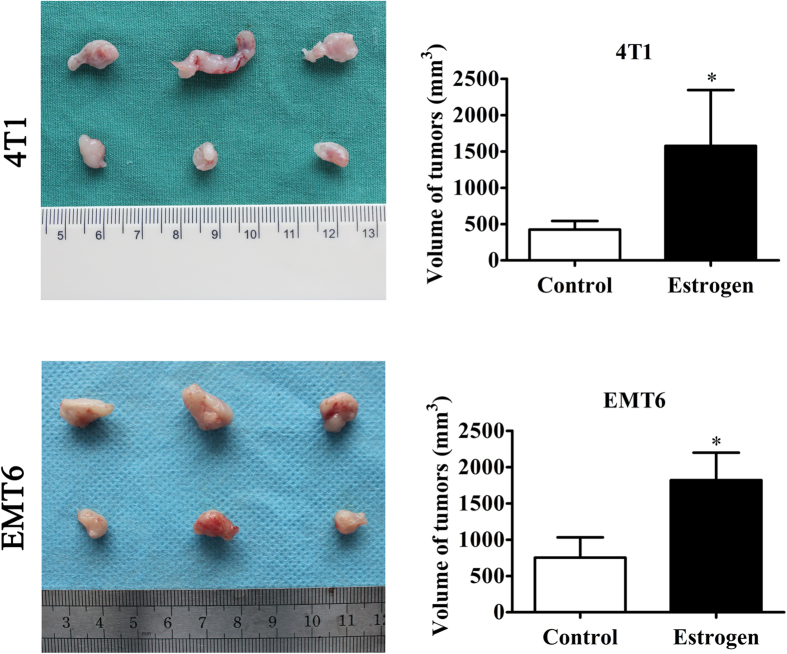
The effect of estrogen on estrogen receptor-negative breast cancer *in vivo*. After bilateral oophorectomy was performed under chlorpromazine anesthesia, the female Balb/c mice were transplanted with either 4T1 cells or EMT6 cells, followed by daily, continuous injections of estrogen (100 μg/kg per day) or vehicle control. Xenografts were harvested after 15 days of growth in the female hosts. The average tumor sizes in each group were evaluated on the 15th day. The values are expressed as the means ± SEM of three independent experiments. *P < 0.05 compared with the control.

The role that estrogen plays in ER-positive BC is well-documented, but the manner in which it contributes to ER-negative breast cancer, which is a more aggressive disease, remains unclear. Evidence shows that regardless of ER status, ovariectomy decreases the long-term risk of BC recurrence in pre-menopausal women, which clearly indicates the involvement of estrogen in ER-negative BC^1^,^3^. Here, we utilized an experimental model of BC that was established by the subcutaneous transplantation of ER-negative murine 4T1 and EMT6 cells into ovariectomized female mice. After the continuous injection of estrogen at a dose of 100 μg/kg per day or the subcutaneous injection of a blank control for two weeks, the mice were sacrificed. The results showed that the tumor sizes in the estrogen group were larger than those in the control group, which indicated that estrogen increased the tumorigenic ability of ER-negative BC cells (Fig. 1).

### Estrogen enhances stromal cell-derived factor 1-α production in cancer-associated fibroblasts

**Figure 2:**
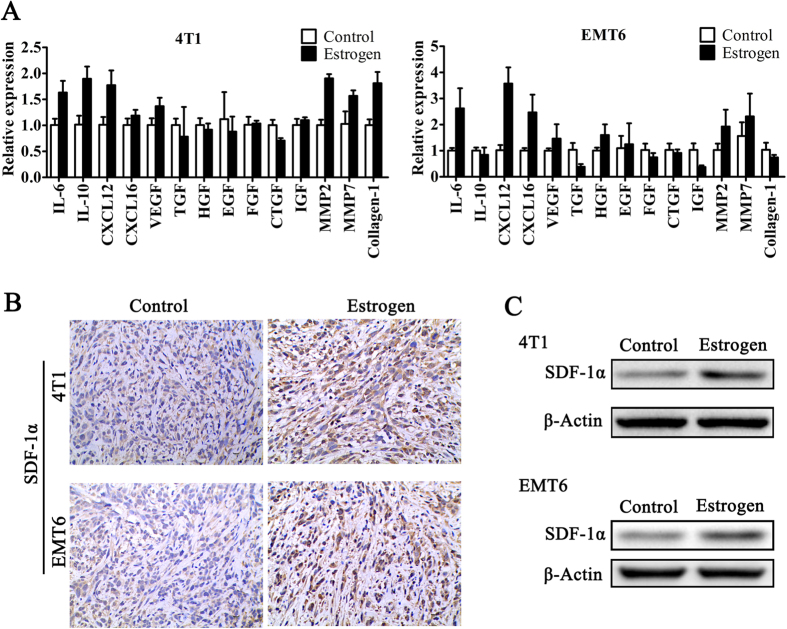
Estrogen increases the expression of SDF-1α in tumor tissue. (**A**) RT-PCR was performed to explore the mRNA levels of several CAF-associated factors in 4T1 and EMT6 xenografts from the estrogen and control groups. (**B**) Immunohistochemistry assays and (**C**) Western blot analysis were used to detect the expression of SDF-1α in 4T1 and EMT6 xenografts from Balb/c mice in these two groups.

**Figure 3:**
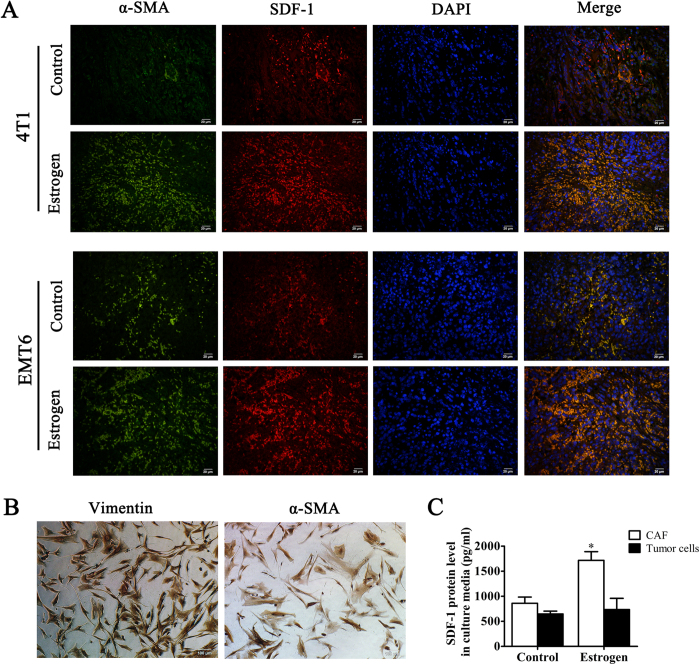
Estrogen enhances the production of stromal cell-derived factor 1 in cancer-associated fibroblasts. (**A**) Co-localization of SDF-1α and α-SMA was detected by immunofluorescence (×100). Nuclei were visualized by DAPI. (**B**) α-SMA and Vimentin, which serve as CAF markers, were used to identify primary cultured fibroblasts by immunocytochemistry (×100). (**C**) CAFs and primary tumor cells were treated with estrogen (10 μM) for 48 h, and the SDF-1α level in the culture media was determined by ELISA. The values are expressed as the means ± SEM of three independent experiments. *P < 0.05 compared with the control.

Estrogen does not influence the proliferation of 4T1 and EMT6 cells. Thus, we focused our attention on the stromal cells in the tumor microenvironment. Fibroblasts are key players in tumorigenesis and constitute the majority of stromal cells within a tumor, especially in breast, prostate, and pancreatic cancers^18^. To elucidate the mechanisms of the different tumorigenic abilities between the estrogen group and the control group, we compared the mRNA levels of several tumor-promoting factors produced by CAFs in 4T1 and EMT6 xenografts^19^,^20^,^21^. After a comprehensive assessment, we found that the levels of SDF-1α (CXCL12) in the estrogen group were significantly increased (Fig. 2A). Furthermore, we found that estrogen could increase the expression of SDF-1α protein in murine tumor tissue (Fig. 2B and C). To determine whether estrogen induces stromal cells in the tumor to produce SDF-1α, we performed immunofluorescence staining using both anti-α-SMA and anti-SDF-1α antibodies on sections of tumor tissue from xenografts of Balb/c mice that received different treatments. Interestingly, the α-SMA protein present in the tumor stroma was largely co-localized with the SDF-1α protein (Fig. 3A). To further investigate the origin of SDF-1α, we collected clinical specimens to obtain CAFs and primary ER-negative BC cells. After the identification of these CAFs by the biomarkers α-SMA and Vimentin (Fig. 3B), we treated CAFs with estrogen for 48 h and detected SDF-1α in the culture media. We found that estrogen could stimulate CAFs to secrete more SDF-1α than the blank control (Fig. 3C). In addition, we used magnetic beads to sort CD326^+^ tumor epithelial cells and found that tumor cells produced less SDF-1α than CAFs regardless of whether they were treated with estrogen (Fig. 3C). Together, these findings indicated that the CAFs were stimulated by estrogen and subsequently produced SDF-1α in the tumor microenvironment.

### SDF-1α recruits MDSCs to the tumor microenvironment

**Figure 4:**
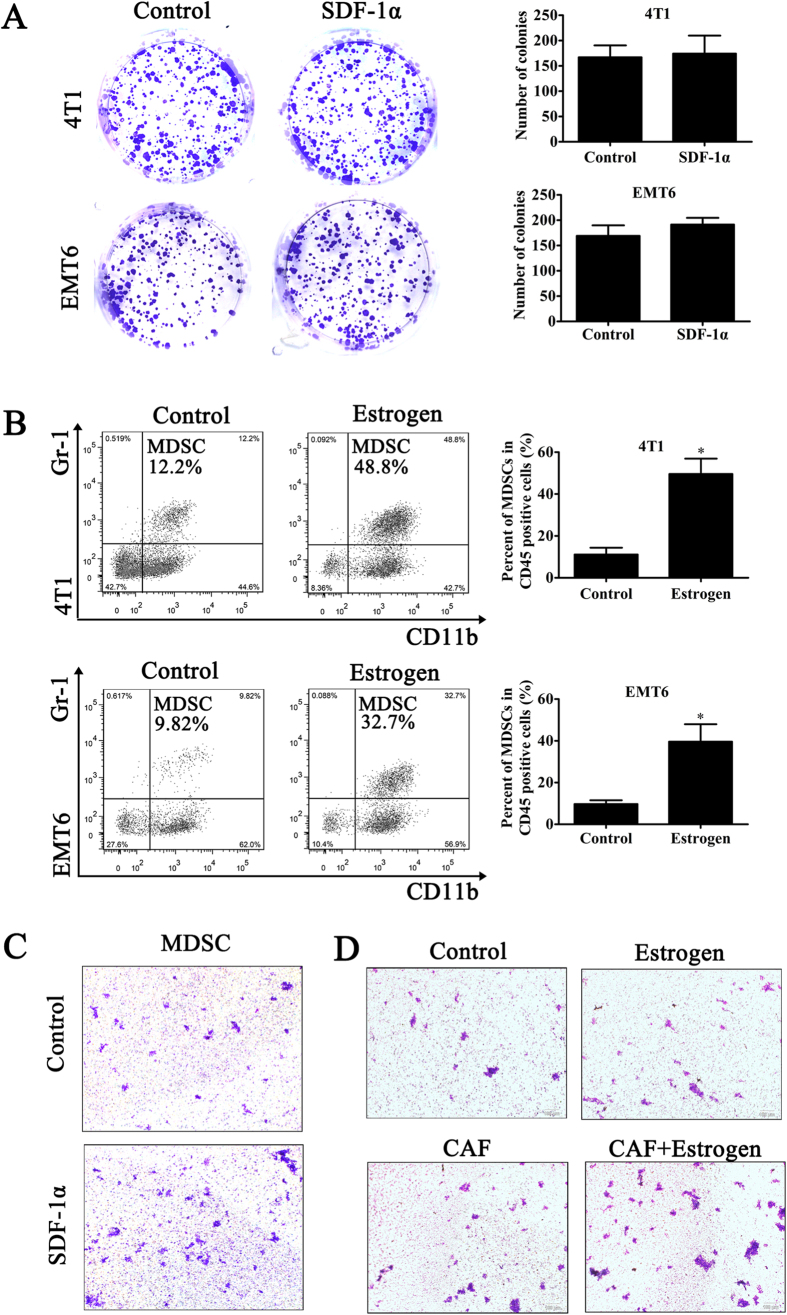
SDF-1α recruits MDSCs to the tumor microenvironment. (**A**) A colony formation assay was used to determine the effect of SDF-1α on the proliferation of 4T1 and EMT6 cells. (**B**) Quantitative analysis of the percentages of MDSCs in a population of CD45^+^ cells in mice from different groups. The values are expressed as the means ± SEM of three independent experiments. *P < 0.05 compared with the control. (**C**) The chemotaxis of MDSCs in response to SDF-1α (100 ng/ml) was measured in chemotaxis chambers (×100). (**D**) CAFs and MDSCs were co-cultured in Transwell chambers to observe the role of estrogen in the recruitment of MDSCs by CAFs that were stimulated by estrogen.

SDF-1α in the tumor microenvironment not only regulates the homeostasis, angiogenesis, proliferation, survival, and migration of cancer cells but also recruits multifunctional immune cells^22^,^23^,^24^. Interestingly, recombinant SDF-1α had no effect on the proliferation of 4T1 and EMT6 cells (Fig. 4A). Thus, we next sought to determine whether SDF-1α plays a functional role in other pathways. Tumor-infiltrating MDSCs in the estrogen and control groups were detected by flow cytometry. The results showed that the number of Gr-1^+^CD11b^+^MDSCs within CD45^+^ cells in tumor tissue from control mice was lower than that in the estrogen-treated mice (Fig. 4B). This result indicated the possibility that the effect of estrogen in the experimental model may depend on the recruitment of MDSCs to the tumor microenvironment. To determine whether SDF-1α can recruit MDSCs, we isolated MDSCs from bone marrow cells using magnetic beads. We then performed a chemotactic assay *in vitro* in a Transwell chamber using recombinant SDF-1α to imitate paracrine SDF-1α. We found few cells in the culture medium in the lower chamber, which indicated the presence of rare MDSCs that had detached from the filter chamber. The results showed that when SDF-1α was present in the lower chamber, the numbers of MDSCs per field were larger than those in the control group; moreover, the MDSCs formed clusters within a field (Fig. 4C). At this point, we had confirmed the interactions between estrogen and SDF-1α, estrogen and MDSCs, and SDF-1α and MDSCs. To further verify the role of estrogen in tumorigenesis, which is dependent on the recruitment of MDSCs via the stimulation of CAFs, we co-cultured CAFs and MDSCs with or without estrogen. The data indicated that the numbers of MDSCs per field were larger when the MDSCs were cultured with both CAFs and estrogen (Fig. 4D). These results signified that the effect of estrogen on ER-negative BC did not directly depend on SDF-1α itself but rather on the recruitment of MDSCs to the tumor microenvironment, where they exert tumor-promoting effects.

### Inhibition of the recruitment of MDSCs may decrease the role of estrogen in ER-negative breast cancer

**Figure 5:**
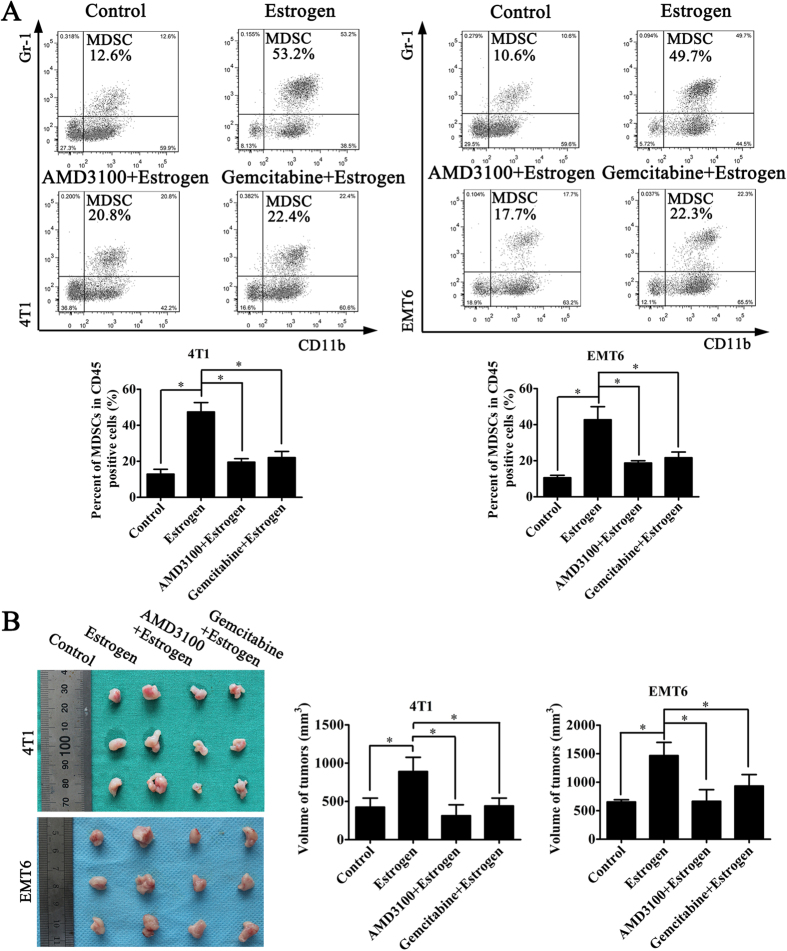
Inhibition of MDSC recruitment neutralizes the effect of estrogen on tumorigenesis. (**A**) AMD3100 was used to block the SDF-1α/CXCR4 axis. After the experimental model was established, we injected the following groups: (1) control group, (2) estrogen group, (3) AMD3100 and estrogen group, and (4) gemcitabine and estrogen group. Quantitative analysis of the percentages of MDSCs in a population of CD45^+^ cells in mice from different groups was performed. The values are expressed as the means ± SEM of three independent experiments. *P < 0.05. (**B**) The average tumor size in each group was evaluated on the 15th day. The values are expressed as the means ± SEM of three independent experiments. *P < 0.05.

To confirm whether the effect of estrogen on ER-negative BC depends on the recruitment of MDSCs induced by SDF-1α *in vivo*, we developed the same animal model as described previously and used AMD3100 to block the SDF-1α/CXCR4 axis or gemcitabine to delete MDSCs. We found that estrogen could increase the number of tumor-infiltrating MDSCs, which could also be inhibited by AMD3100 and gemcitabine (Fig. 5A). Furthermore, when the SDF-1α/CXCR4 axis was blocked by AMD3100 or if MDSCs were deleted with gemcitabine, the effect of estrogen on tumorigenesis was neutralized (Fig. 5B). Together, these findings indicated that the estrogen-induced secretion of SDF-1α can recruit MDSCs into the tumor microenvironment, where they exert tumor-promoting effects.

## Discussion

While the roles of estrogen in ER-positive BC are well documented, the contributions of estrogen to ER-negative BC are unclear. To study the mechanism by which estrogen promotes the development of ER-negative BC, we established a tumor-bearing mouse model by the subcutaneous inoculation of 4T1 and EMT6 murine BC cells into ovariectomized mice. We then treated the mice with exogenous estrogen to detect its role in ER-negative BC. This experimental model avoids the influence of endogenous estrogen and allows for better separation and control of the different groups.

Estrogen does not affect the proliferation of some ER-negative cancer cells *in vitro* or the growth of primary tumors *in vivo*^25^. In this study, we found that estrogen promoted tumorigenicity when ER-negative BC cells were transplanted into mice in an experiment model. This result seems to conflict with the lack of clinical responses of ER-negative tumors to endocrine therapy. In fact, evidence has suggested that estrogen can increase the systemic capacity for angiogenesis, stromalization, and bone marrow cell recruitment, and this mechanism is partly responsible for the promotion of tumorigenesis of ER-negative tumors^5^. Thus, estrogen can increase tumorigenicity through one pathway and not directly influence the proliferation of ER-negative cancer cells. Therefore, unlike ER-positive BC, endocrine treatment is not a direct and effective treatment for ER-negative BC. In addition, the promotion of tumorigenesis of ER-negative BC by estrogen may be an auxiliary mechanism and may occur in the early stages of tumor development. Therefore, it is understandable why ER-negative BC do not exhibit clinical responses to endocrine therapy, and this is not contradictory to the notion that estrogen promotes the tumorigenesis of ER-negative BC.

Here, we focused on the stromal cells in the tumor microenvironment. The SDF-1α produced by cancer-associated fibroblasts enhances tumor growth by direct paracrine stimulation^12^. In tumor tissue, we observed a higher level of SDF-1α in the estrogen group. Furthermore, we found that the fibroblast-like cells that expressed SDF-1α were also positive for α-SMA. At the same time, an enzyme-linked immunosorbent assay (ELISA) indicated that estrogen could simulate CAFs, but not primary BC cells, to produce SDF-1α. Thus, it is likely that estrogen can stimulate CAFs to secrete SDF-1α in this animal model. We note, however, that recombinant SDF-1α exhibits no effect on the proliferation of 4T1 and EMT6 cells. These lines of evidence support the idea that estrogen facilitates the progression of ER-negative BC through CAF-secreted SDF-1α in the tumor microenvironment, but this estrogen-induced SDF-1α does not influence the cancer cells directly.

MDSCs, which are considered immature myeloid cells, include precursors of dendritic cells, macrophages, and granulocytes. Elevated numbers of MDSCs were found to cause global and profound immune suppression in many cancer patients and tumor-bearing animals^26^. The accumulation of MDSCs within tumor tissue is driven by several chemokines produced by tumor-associated stromal cells^27^. Here, we found that tumors in mice in the estrogen group had higher numbers of MDSCs. We also determined that recombinant SDF-1α mediated the recruitment of MDSCs *in vitro*. When CAFs and MDSCs were co-cultured with estrogen, the numbers of recruited MDSCs were larger. An explanation for these findings is that estrogen stimulates CAFs to secrete high levels of SDF-1α to attract MDSCs, which play a crucial role in immune evasion and limit the effectiveness of the immune response.

MDSCs function in immune suppression and secrete several pro-tumor factors such as nitric oxide (NO), reactive oxygen species (ROS), vascular endothelial growth factor (VEGF), and matrix metalloproteinase 9 (MMP9), all of which contribute to tumor progression^28^. In this study, estrogen caused the accumulation of MDSCs in the tumor microenvironment, and this effect could be repressed by AMD3100 or gemcitabine in our experiment model. When the recruitment of MDSCs was inhibited by AMD3100 or gemcitabine, the promotion of tumorigenesis by estrogen was suppressed. Here, AMD3100 is a receptor antagonist for CXCR4 to reduce the effect of SDF-1α on the recruitment of MDSCs, and gemcitabine is an agent that can eliminate MDSCs directly. Thus, we confirmed that estrogen has tumor-promoting functions depending on the recruitment of MDSCs, which occurs through the secretion of SDF-1α by CAFs.

In conclusion, the progression of ER-positive and ER-negative BC can be accelerated by estrogen. In ER-negative BC, estrogen does not exert an influence on cancer cells through the classical pathway; instead, it promotes the secretion of SDF-1α by tumor stromal cells. The SDF-1α/CXCR4 axis recruits MDSCs into the tumor microenvironment, where they function in immune evasion and exert tumor-promoting effects. Here, we offered a new perspective to improve our understanding of the role of estrogen in ER-negative BC. Thus, the inhibition of the SDF-1α/CXCR4 signaling pathway or the recruitment of MDSCs to the tumor microenvironment may attenuate the progression of ER-negative BC.

## Materials and Methods

### Ethics statement

All animal experiments were performed in accordance with the guidelines set forth by the Chinese National Institutes of Health and were approved by the Ethics Committee of Tongji Medical College, Huazhong University of Science and Technology. All clinical tissues were obtained from patients with breast carcinoma who were seen at Union Hospital (WuHan, China) according to the approved guidelines of the hospital ethics committee. Written informed consent was obtained from all subjects.

### Cell culture

Murine 4T1 cells and EMT6 cells were obtained from American Type Culture Collection (ATCC, USA) and were cultured in RPMI-1640 medium (Hyclone, USA) supplemented with 10% fetal bovine serum (ScienCell, USA) and 1% penicillin/streptomycin.

CAFs were isolated from cancer-associated regions of whole breast tissues. The dissociated tissues were minced with scissors and digested with collagenase type I (1 mg/ml; Sigma) and hyaluronidase (100 U/ml; Sigma) at 37 °C for 12 h in Dulbecco’s Modified Eagle’s Medium (DMEM) supplemented with 2% bovine serum albumin (BSA). The stromal cell-enriched supernatant was collected for centrifugation at 250 g for 10 min. The cell pellet was resuspended in DMEM/F12 medium supplemented with 10% fetal bovine serum, 10 ng/ml EGF (Peprotech, USA), and 10 ng/ml FGF (Peprotech, USA). We plated the cell suspension in cell culture flasks, which were left undisturbed for 2 to 5 days.

Primary BC cells were sorted using CD326 (EpCAM) MicroBeads (Miltenyi Biotech, Germany), according to the manufacturer’s instructions, for the positive selection of viable epithelial tumor cells from single-cell preparations from tissues of patients with breast carcinoma. The positively selected cells were suspended in DMEM/F12 medium supplemented with 10% fetal bovine serum.

All cell lines were cultured in a humidified incubator at 37 °C and 5% CO_2_.

### Surgery in the tumor-bearing mice

Female Balb/c mice (4 weeks of age) were obtained from the Experimental Animal Center of Wuhan University (Wuhan, China) and housed in the Experimental Animal Center of Tongji Medical College (Wuhan, China). All female Balb/c mice underwent bilateral oophorectomy under chlorpromazine anesthesia. After a week-long recovery, the Balb/c mice were injected subcutaneously with one million 4T1 cells and EMT6 cells resuspended in PBS. During the next two weeks, mice in the different groups were given a subcutaneous injection of (1) PBS (blank control group), (2) estrogen (100 μg/kg, Sigma Aldrich, USA), (3) estrogen and AMD3100 (5 mg/kg, Sigma Aldrich, USA), or (4) estrogen and gemcitabine (Sigma Aldrich, USA). Mice in the first three groups were injected every day. Mice in the final group were given a intraperitoneal injection of a single dose of 100 mg/kg gemcitabine one day before the cells were transplanted, and estrogen was injected every day thereafter as previously described. The tumor tissues were obtained from tumor-bearing mice after they were sacrificed.

### Real-time PCR

Total RNA was extracted from tissues using TRIzol (Invitrogen, USA) according to the manufacturer’s protocol. All mRNA was reverse-transcribed according to the protocol of the PrimeScript^®^ RT Master Mix (TaKaRa, Japan) and was used in quantitative reverse transcription-polymerase chain reaction (qRT-PCR) analysis with SYBR Premix Ex Taq (TaKaRa, Japan) in a StepOnePlus™ Real-time PCR system (Applied Biosystems, USA). The PCR conditions were as follows: 30 s at 95 °C, followed by 40 cycles at 95 °C for 5 s and 60 °C for 30 s. All reactions were performed in triplicate. The expression of mRNAs was defined according to the threshold cycle (Ct), while the relative expression of mRNAs was calculated using the comparative CT (2^−ΔΔCT^) method. The primer pairs used to measure the levels of each gene are listed in Supplementary Table S1.

### Immunohistochemistry (IHC)

Sections were deparaffinized in xylene, rehydrated in a diluted alcohol series, and immersed in 0.3% H_2_O_2_ in methanol to quench endogenous peroxidase activity. Sections were then subjected to antigen retrieval in sodium citrate buffer at 95–100 °C. To reduce non-specific staining, each section was treated with 4% bovine serum albumin in PBS with 0.1% Tween 20 (PBST) for 30 min. The sections were then incubated with anti-SDF-1α (1:50; ImmunoWay Biotechnology Company, USA) in PBST overnight at 4 °C. The next day, the sections were incubated with a peroxidase-conjugated goat anti-rabbit IgG antibody for 30 min at 37 °C. After washing in PBS, the tissue sections were stained with diaminobenzidine, counterstained in hematoxylin, and examined under a light microscope.

### Western blot analysis

Tumor tissues were minced with scissors and lysed in RIPA buffer (50 mM Tris pH 7.4, 150 mM NaCl, 1 mM EDTA, 1% Triton X-100, 1% Na-Doc, 0.1% SDS) supplemented with a protease inhibitor cocktail (Roche) on ice for 30 min. After denaturation, the proteins were separated by 12% sodium dodecyl sulfate-polyacrylamide gel electrophoresis (SDS-PAGE) and then transferred to polyvinylidenedifluoride (PVDF) membranes (Millipore, USA). After blocking with 5% nonfat milk and 0.1% Tween 20 in TBS for 1 h, the PVDF membranes were probed with primary antibodies at 4 °C overnight, followed by incubation with goat anti-rabbit secondary antibodies at room temperature for 1 h. The primary antibodies used in this analysis were SDF-1α (ImmunoWay Biotechnology Company, USA) and β-Actin (Boster, China). The protein signals were detected by the enhanced chemiluminescence (ECL) method.

### Immunofluorescence staining

The co-expression of SDF-1α (Santa Cruz Biotechnology, USA) and α-SMA (Abcam, USA) in the tissue sections was analyzed by immunofluorescence. Briefly, tissue sections (5 μm in thickness) were deparaffinized and then subjected to antigen retrieval in sodium citrate buffer at 95–100 °C. The slides were incubated with the primary antibodies (1:50 dilution) at 4 °C overnight, which was followed by incubation with PE-conjugated anti-goat and FITC-conjugated anti-rabbit secondary antibodies for 1 h at room temperature. Finally, these slides were mounted with Prolong Gold containing 4′, 6-diamidino-2-phenylindole (DAPI, Boster, China). Fluorescent images were obtained using an Olympus IX71 epifluorescence microscope (Olympus, Japan).

### Identification of fibroblasts

Primary cultured cancer-associated fibroblasts derived from human BC were confirmed by immunocytochemistry using a mouse anti-α-SMA monoclonal antibody (1:100; Abcam, USA) and a rabbit anti-Vimentin polyclonal antibody (1:100; Cell Signaling Technology, USA). Briefly, the cells were seeded at a density of 1 × 10^5^ cells/well in 12-well culture plates. After incubation for 24 h at 37 °C and 5% CO_2_, the cells were fixed in 4% formalin for 15 min and permeabilized in 0.1% Triton X-100 for 5 minutes. To reduce non-specific staining, the cells were treated with 4% bovine serum albumin in PBS with 0.1% Tween 20 for 30 min. The cells were then incubated with an anti-α-SMA monoclonal antibody and an anti-Vimentin antibody overnight at 4 °C. After three successive rinses in PBS, the cells were incubated for 15 min with avidin-biotin horseradish peroxidase complex, and the reaction was visualized using 0.02% 3,3′-diaminobenzidine tetrahydrochloride as the chromogen in Tris-HCl buffer. Hematoxylin was used to counterstain the nuclei.

### ELISA

Cancer-associated fibroblasts and primary BC cells derived from human BC were seeded separately in 12-well plates. After treatment with estrogen (10 μM) in DMEM for 48 h, the quantification of the SDF-1α level in the supernatants of fibroblasts and tumor cells was performed by ELISA according to the protocol of a commercially available human SDF-1α ELISA kit (NeoBioscience, China). Three wells per condition were tested, and all experiments were repeated in triplicate.

### Colony formation assay

The colony formation assay was performed for 4T1 cells and EMT6 cells. Briefly, single cells (1,500 cells per well) were plated in 6-well plates and cultured with or without SDF-1α (100 ng/ml) for 2 weeks. The culture medium was replaced every three days. After they were stained with crystal violet, the cells were photographed and analyzed for colony formation efficiency. The experiments were performed in triplicate.

### Isolation of MDSCs

Myeloid-derived suppressor cells were isolated from bone marrow cells by magnetic bead sorting using the MACS system (Miltenyi Biotech, Germany). Bone marrow cells were obtained from the femurs and tibias of female Balb/c mice using a syringe. According to the manufacturer’s instructions, positive selection of Gr-1^high^Ly-6G^+^ was performed with LS columns. After the first depletion of Ly-6G^+^ cells using LS columns, Gr-1^dim^Ly-6G^-^ cells were enriched, and subsequent positive selection of Gr-1^+^ cells was performed using MS columns. The isolated cells were identified by flow cytometry using PerCP/cy5.5-labeled anti-CD45, PE-labeled anti-CD11b, and APC-labeled anti-Gr-1 (Biolegend, USA).

### Flow cytometry

To investigate tumor-infiltrating MDSCs, freshly resected tumors from 4T1 and EMT6 tumor-bearing mice were cut into pieces and were then enzymatically digested in DMEM medium containing collagenase type I and hyaluronidase with 2% bovine serum albumin for 3 h at 37 °C. The tissue was subjected to gentle shaking every 10 min until all the tumor tissue had resolved into a cell suspension. The cell suspension was filtered through 40-μl cell strainers (BD Biosciences) to remove the undigested pellet. After centrifugation, the cells were stained by immunofluorescence using anti-CD45, anti-CD11b, and anti-Gr-1 antibodies and then analyzed by flow cytometry.

### Chemotaxis assay

Chemotaxis assays were performed using Transwell chambers (8-μm pore size; Corning, USA). MDSCs were isolated from the bone marrow of female Balb/c mice by magnetic beads. MDSCs were seeded into the upper chamber at a density of 1 × 10^5^ cells per well in 200 μl of serum-free medium, and serum-free medium containing 100 ng/ml SDF-1α was used as a chemotactic agent in the lower chamber. After the cells were cultured for 24 h at 37 °C, the cells on the membranes were fixed in 4% paraformaldehyde and stained with 0.1% crystal violet. The upper surface of the Transwell membrane was wiped gently with a cotton swab, and the chambers were analyzed using a bright-field microscope.

Briefly, with regard to the co-culture of CAFs and MDSCs, the CAFs were obtained in the same manner as human CAFs from the xenografts of Balb/c mice. They were pre-seeded in the lower chambers and treated with estrogen (10 μM) for 48 h. MDSCs isolated from the bone marrow of female Balb/c mice were seeded in the upper chamber. MDSCs cultured for 24 h with no estrogen, with estrogen, or with CAFs alone in the lower chamber served as the control. All experiments were performed in triplicate.

### Statistical analysis

Each experiment was performed in at least three independent trials. The results are presented as the means ± SEM. Statistical analyses were performed using Student’s t-test, and values of P < 0.05 were considered statistically significant.

## Additional Information

**How to cite this article:** Ouyang, L. *et al*. Estrogen-induced SDF-1α production promotes the progression of ER-negative breast cancer via the accumulation of MDSCs in the tumor microenvironment. *Sci. Rep.* **6**, 39541; doi: 10.1038/srep39541 (2016).

**Publisher's note:** Springer Nature remains neutral with regard to jurisdictional claims in published maps and institutional affiliations.

## Supplementary Material

Supplementary Table S1
