# Supplementary material for: Estrogen-induced SDF-1α production promotes the progression of ER-negative breast cancer via the accumulation of MDSCs in the tumor microenvironment
Source: Sci Rep. 2016 Dec 20;6:39541. doi: 10.1038/srep39541 (PMC5172230; doi:10.1038/srep39541)
Supplement: Supplementary Table S1 [file srep39541-s1.doc]

**Title:** Estrogen-induced SDF-1α production promotes the progression of ER-negative breast cancer via the accumulation of MDSCs in the tumor microenvironment

**Authors:** Liquan Ouyang, Weilong Chang, Bin Fang, Jieting Qin, Xincai Qu & Fanjun Cheng

**Table S1: Pr**imer names and sequences.

| **Primer Name** | **Forward primer Sequence (5’ to 3’)** | **Reverse primer Sequence (5’ to 3’)** |
| --- | --- | --- |
| IL-6 | GAGGATACCACTCCCAACAGACC | AAGTGCATCATCGTTGTTCATACA |
| IL-10 | GCTCTTACTGACTGGCATGAG | CGCAGCTCTAGGAGCATGTG |
| CXCL12 | CACCTCGGTGTCCTCTTG | GGTCAATGCACACTTGTCTG |
| CXCL16 | CCAGTGGGTCCGTGAACT | CTCGTGTCCGAAGGTGTC |
| VEGFα | CGAGATAGAGTACATCTTCAAGC | TTGATCCGCATGATCTGCATGG |
| TGF-β | TCCCTCAACCTCAAATTATTCA | GCGGTCCACCATTAGCAC |
| HGF | CCCCTATGCAGAAGGACAGAA | GCCCTGTTCCTGATACACC |
| EGF | ATCCCATCGGTAATAAGG | CAGCCTCCATTCCTGTAT |
| FGF21 | CTACCAAGCATACCCCATCC | GCCTACCACTGTTCCATCCT |
| CTGF | CTTCTGCGATTTCGGCTCC | TACACCGACCCACCGAAGA |
| IGF-1 | GCAGATGTAGTCTTGCCCTTGT | CAGCGTGCCTGATGTTCC |
| MMP2 | CCCCGATGCTGATACTGA | CTGTCCGCCAAATAAACC |
| MMP7 | AGAATACTCACTAATGCCAAAC | TTCACTCCTGCGTCCTCA |
| Collagen-1 | ATGTTCAGCTTTGTGGACCTC | TCCCTCGACTCCTACATCTTC |
| GAPDH | AGGTCGGTGTGAACGGATTTG | TGTAGACCATGTAGTTGAGGTCA |
